# Scale-Aware Transformers for Diagnosing Melanocytic Lesions

**DOI:** 10.1109/ACCESS.2021.3132958

**Published:** 2021-12-06

**Authors:** WENJUN WU, SACHIN MEHTA, SHIMA NOFALLAH, STEVAN KNEZEVICH, CAITLIN J. MAY, OLIVER H. CHANG, JOANN G. ELMORE, LINDA G. SHAPIRO

**Affiliations:** 1 Department of Medical Education and Biomedical Informatics, University of Washington, Seattle, WA 98195, USA; 2 Department of Electrical and Computer Engineering, University of Washington, Seattle, WA 98195, USA; 3 Pathology Associates, Clovis, CA 93611, USA; 4 Dermatopathology Northwest, Bellevue, WA 98005, USA; 5 Department of Pathology, University of Washington, Seattle, WA 98195, USA; 6 David Geffen School of Medicine, UCLA, Los Angeles, CA 90024, USA; 7 Paul G. Allen School of Computer Science and Engineering, University of Washington, Seattle, WA 98195, USA

**Keywords:** Convolutional neural network, histopathological images, melanocytic risk lesions, melanoma, multi-scale, transformers, skin cancer diagnosis, whole-slide image classification

## Abstract

Diagnosing melanocytic lesions is one of the most challenging areas of pathology with extensive intra- and inter-observer variability. The gold standard for a diagnosis of invasive melanoma is the examination of histopathological whole slide skin biopsy images by an experienced dermatopathologist. Digitized whole slide images offer novel opportunities for computer programs to improve the diagnostic performance of pathologists. In order to automatically classify such images, representations that reflect the content and context of the input images are needed. In this paper, we introduce a novel self-attention-based network to learn representations from digital whole slide images of melanocytic skin lesions at multiple scales. Our model softly weighs representations from multiple scales, allowing it to discriminate between diagnosis-relevant and -irrelevant information automatically. Our experiments show that our method outperforms five other state-of-the-art whole slide image classification methods by a significant margin. Our method also achieves comparable performance to 187 practicing U.S. pathologists who interpreted the same cases in an independent study. To facilitate relevant research, full training and inference code is made publicly available at https://github.com/meredith-wenjunwu/ScATNet.

## I. INTRODUCTION

Invasive melanoma, with more than 100,000 estimated new cases in 2021, is one of the most commonly diagnosed cancers in the U.S [1]. The ‘‘gold standard’’ for diagnosis of skin biopsy specimens relies on the visual assessments of pathologists. Unfortunately, diagnostic errors are common, and even expert pathologists may not reach consensus on diagnostically challenging cases in many areas within pathology [2]–[5]. For instance, pathologists disagree in up to 60% of melanoma in situ and stage T1a invasive cases [6]. Variability in diagnostic decisions is a serious problem and can cause substantial patient harm. A computer-aided diagnostic system can act as a *second reader* and help pathologists reduce classification uncertainties.

**FIGURE 1.**
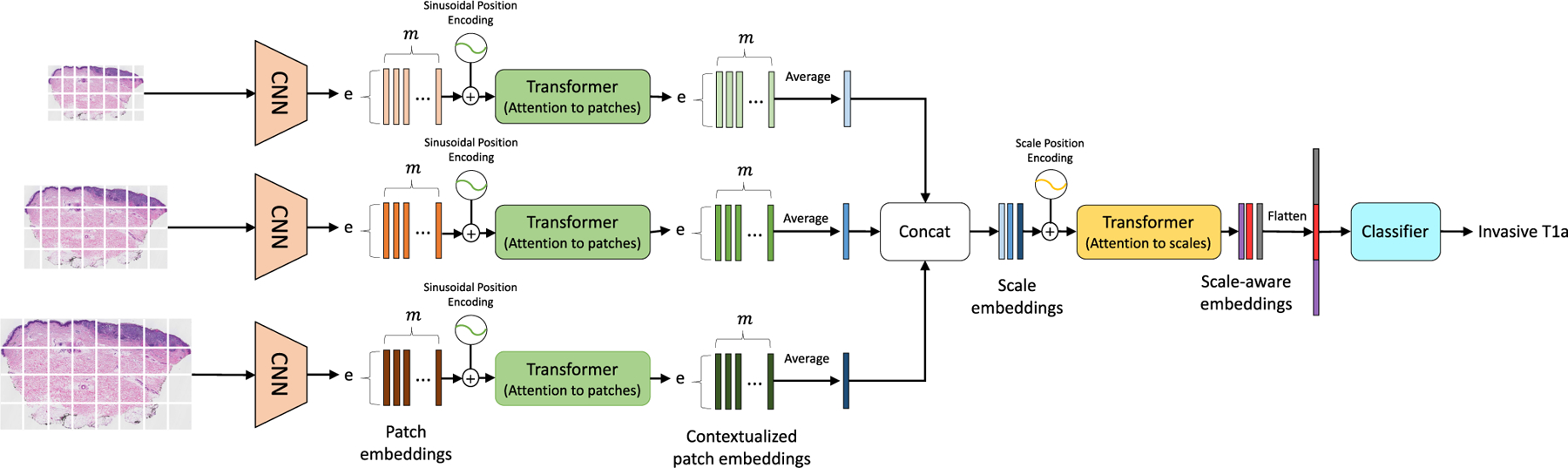
Overview of ScATNet for classifying skin biopsy images. To learn representations from these large WSIs at multiple input scales in an end-to-end fashion, ScATNet factorizes the classification pipeline into three steps. The first step involves learning local patch-wise embeddings using an off-the-shelf CNN for each input scale independently. In the second step, ScATNet learns inter-patch representations using transformers and produces contextualized patch embeddings for each input scale. In the last step, ScATNet learns inter-scale representations from concatenated multi-scale contextualized patch embeddings using another transformer network and produces scale-aware embeddings, which are then classified linearly into diagnostic categories.

For a reliable diagnostic system, it is important to obtain representations that reflect both the content and context of the input biopsy image. This paper introduces a self-attention-based deep neural network called the Scale-Aware Transformer Network (ScATNet) for classifying melanocytic skin lesions in digital whole slide images (WSIs). ScATNet, shown in Figure 1, extends the standard transformer model of Vaswani *et al.* (2017) to learn representations from biopsy images at multiple input scales. The key idea is to learn patch-wise representations independently for each input scale using a convolutional neural network (CNN), and then learn inter-patch and inter-scale representations from concatenated multi-scale contextualized patch embeddings using transformers. This allows our system to learn diagnostic class-specific representations at different scales and helps improve the performance. Also, each WSI contains multiple tissue slices, while usually only one or two tissue slices help pathologists in diagnosis. We introduce a soft-label assignment method to (1) reduce the ambiguity between different tissue slices in a WSI and (2) improve the diagnostic classification performance.

We demonstrate the effectiveness of ScATNet on a skin biopsy image dataset [6]. Experimental results show that ScATNet outperforms state-of-the-art methods by a significant margin. For example, ScATNet is 8% more accurate than the method proposed by Chikontwe *et al.* [7] and 6% more accurate than the method proposed by Hashimoto *et al.* [8]. Importantly, ScATNet delivers comparable performance to 187 practicing pathologists who interpreted the same test set cases in an independent study.

To summarize, the main contributions of this paper are: (1) a novel self-attention-based end-to-end framework for classifying WSIs at multiple input scales (Section III-B), (2) a soft label assignment method to reduce ambiguities that arise by assigning the same label to all tissue slices in a WSI (Section III-C), and (3) experimental results, along with comparisons with state-of-the-art methods and practicing U.S. pathologists, demonstrating ScATNet’s competitive performance (Section IV).

## II. RELATED WORK

ScATNet was inspired by the success of several works in the area of WSI image classification and transformers. We briefly discuss these approaches in the following sub-sections.

### A. MULTIPLE INSTANCE LEARNING (MIL)

Convolutional neural networks (CNNs) are the de facto machine learning-based method for image classification, including WSIs [9]–[11]. Unlike the images in standard datasets (e.g., ImageNet [12]), WSIs are orders of magnitude larger and cannot be processed in an end-to-end fashion using CNNs. The MIL framework has been widely studied for classifying different types of WSIs, such as lung [11], kidney [13], and breast [14]. In general, the input WSI is divided into instances (or patches) and the same classification label is assigned to all instances during training. During evaluation, methods such as averaging and majority voting are used to aggregate the information from all instances in an image and produce an image-level classification label. Though these approaches are effective, they learn local instance-wise representations. This work extends the MIL framework with the transformers of Vaswani *et al.* (2017) to learn global representations in an end-to-end fashion. In our experiments, we compared our method to the MIL methods of Chikontwe *et al.* [7] and Hashimoto *et al.* [8]. In addition, we compared our system to a standard patch-based CNN classification framework. Details of these methods are described in section IV-D.

### B. PATCH-BASED FEATURE AGGREGATION

Patch-based methods provide a solution to the gigapixel size of WSIs, while only requiring slide-level labels. However, learning robust instance representations is challenging due to the ambiguity in instance-level labels. To address this, many recent methods [11], [15] adopt a two-step approach that consists of (1) training an instance encoder for obtaining a prediction score or low-dimensional features, and (2) learning a model that aggregates the features extracted by the learned instance encoder to form instance-level information for slide-level prediction. Although this approach has had some success, it often suffers from worse performance when noisy labels are present, causing the features to not be representative of their given labels. In our experiments, we compared our method with a CNN-based deep-feature-aggregation framework developed by Mercan *et al.* [15]. Details of this method are described in section IV-D.

### C. SEGMENTATION-BASED METHODS

These approaches use semantic information about tissues in a WSI to produce an image-level decision [16]–[20]. Typically, these approaches have three steps: (1) produce a tissue-level semantic segmentation mask using CNNs for an input WSI, (2) extract features, such as distribution of tissues, from these semantic masks, and (3) produce an image-level decision using the features extracted from the semantic masks. These approaches learn global representations (information from segmentation masks) and have been found to be more effective than plain patch- and MIL-based approaches. However, one key challenge with these approaches is that they require tissue-level segmentation masks whose collection is challenging, because (1) domain experts are required for annotations and (2) pixel-wise annotations on images of gigapixel order is very time consuming. In contrast, this work introduces a method for learning global representations from histopathological WSIs without the need for tissue-level segmentation masks.

### D. END-TO-END LEARNING

Recent attempts at WSI classification focus on designing a single neural network that aggregates information from the entire image in a single shot [21], [22]. These methods extend the MIL-based approach with gradient check-pointing and advanced feature-fusion methods, such as self-attention. Inspired by model-level parallelism [9] and gradient check-pointing [23], these approaches break down the WSI classification pipeline into multiple stages and cache the intermediate results of CNN layers during forward and backward passes, allowing the systems to learn representations in an end-to-end fashion. For example, Mehta *et al.* [21] uses the transformers of Vaswani *et al.* (2017) to aggregate the information from all instances in a breast biopsy image, while Pinckaers *et al.* [22] stitches the instance-wise feature maps of a prostate cancer image at a very low-spatial resolution obtained from a CNN to produce an image-level feature map. ScATNet extends these approaches for classifying skin biopsies. Unlike these approaches that use WSIs at a single scale (typically at a zoom-level of 10×) for classification, this work proposes a scale-aware transformer that adapts to and uses the representations from multiple input scales to achieve higher classification performance. In our experiments, we compared our method with a CNNbased end-to-end WSI classification framework developed by Pinckaers *et al.* [22], details of this which are described in section IV-D.

### E. VISION TRANSFORMERS

The transformers of Vaswani *et al.* [24], initially introduced for the task of machine translation (e.g., [25], [26]), are being explored for modeling images and computer vision tasks (e.g., [27], [28]). Transformers use self-attention, which allows the inputs (e.g., words in a sentence) to interact with each other and learn global representations. Carion *et al.* [29] extended the standard encoder-decoder network of Vaswani *et al.* [24] for the task of object detection. Recent work has extended transformers using a patch-based approach to image recognition at a large scale [27], [28]. Concurrent work has also utilized transformers and self-attention to medical image segmentation [30]–[33] and classification [34].

Motivated by (1) the success of transformers in vision, (2) the methods for learning representations from different input scales [35]–[37], and (3) the importance of input scales for diagnosis in clinical settings [38], [39], we propose a scale-aware transformer model that adapts to the information from different input scales using self-attention and predicts the classification label.

## III. METHOD

This section first reviews the architecture of transformers and then elaborates on the details of the proposed method, scale-aware transformers (Section III-B), that allows our system to learn representations from histopathological images at multiple scales in an end-to-end fashion. In Section III-C, a soft-labeling method is discussed that reduces the ambiguity in instance-level (patches) labels and improves the learning of representations from skin-biopsy images. The software associated with this work will be made available.

### A. TRANSFORMERS

**FIGURE 2.**
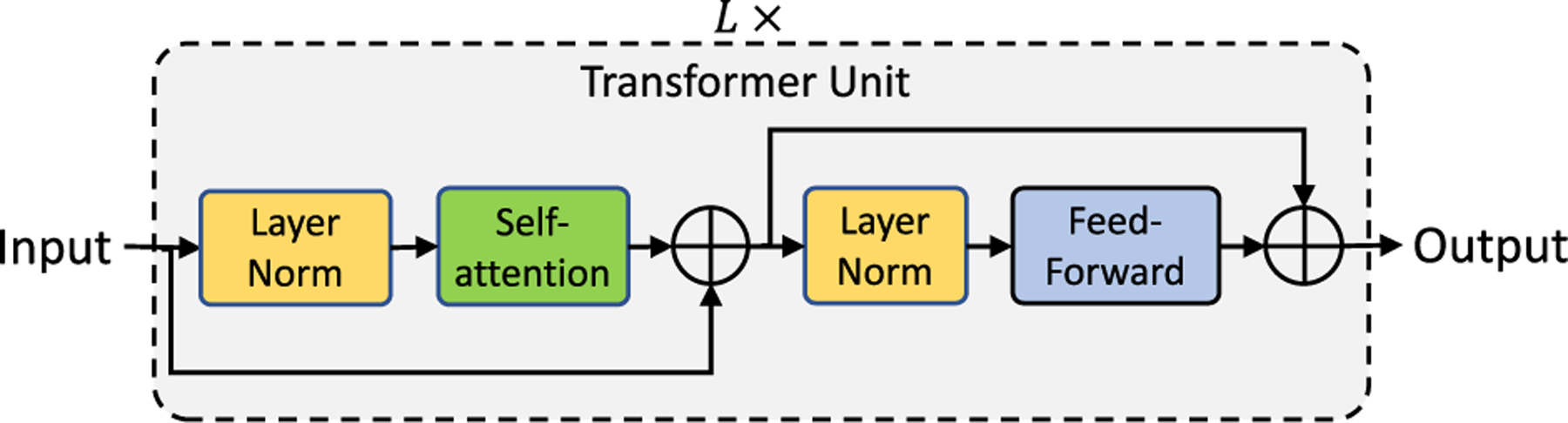
The transformer network stacks *L* transformer units sequentially. Each transformer unit consists of self-attention and feed-forward modules.

The transformer unit, shown in Figure 2, is comprised of two modules: (1) self-attention and (2) feed-forward. The self-attention module allows the inputs to interact with each other and learn contextual relationships. This layer applies three projections, with each projection branch having multiple linear layers to the input $\text{I}\in {\mathbb{R}}^{n\times e}$ to produce query (**Q**), key (**K**), and value (**V**) embeddings, where *n* is the number of inputs and *e* is the input dimensionality. A dot-product between query (**Q**) and key (**K**) is computed to produce an *n*×*n* matrix to which a row-wise softmax is applied to encode relationships between the *n* inputs. Finally, a weighted sum is computed between the resultant *n* × *n* matrix and *V*.


(1) $$\text{Self-attention}\left(\text{Q},\text{K},\text{V}\right)=\text{softmax}\left(\left(\text{Q}\cdot {\text{K}}^{T}\right)\cdot \text{V}\right)$$

The feed-forward module stacks two linear layers, and is responsible for learning wider representations. The first linear layer projects the input to a high-dimensional space, while the second linear layer projects from the high-dimensional space to the same dimensionality as that of the input. This work extends the transformers model to learn scale-aware representations from skin biopsy images.

### B. SCALE-AWARE TRANSFORMERS

Patch-based CNNs are state-of-the-art WSI classification methods that allow computer systems to learn representations from gigapixel size images (e.g. [11], [13], [14], [16], [40]). One of the main limitations of such systems is that they learn local representations, since the context capturing ability of such systems is limited to the patch-level. Another challenge is learning representations from multiple input scales. Because of limited GPU memory and the sheer size of these images, training multi-scale classification systems is computationally intractable. For example, the average size of a WSI (11K × 9.5K) in our dataset is 2000 times larger than the standard image classification dataset: the ImageNet [41] (224 × 224).

Motivated by the recent advancements in computer vision, especially vision transformers and the importance of input scales in clinical settings, this paper introduces scale-aware transformers in ScATNet, which allows our system to learn local and global representations from multiple input scales in an end-to-end fashion. Figure 1 shows the overview of ScATNet, which has three main steps: (1) learn local patch-wise embeddings using a CNN for each input scale, (2) learn contextualized patch-embeddings for each input scale using transformers, and (3) learn scale-aware embeddings across multiple input scales using transformers. These steps are described below.

#### 1) PATCH EMBEDDINGS

The input WSI image ${\text{X}}^{sc}\in {\mathbb{R}}^{W\times H}$ at scale *sc* with width *W* and height *H* is divided into *m* non-overlapping patches ${\text{X}}^{sc}=\left({\text{x}}_{1}^{sc},\ldots ,{\text{x}}_{m}^{sc}\right)$, where ${\text{x}}_{i}^{sc}$ is the *i*-th patch with width $\frac{W}{\sqrt{m}}$ and height $\frac{H}{\sqrt{m}}$. Patch-wise feature representations, referred to as patch embeddings, are obtained using an off-the-shelf CNN. The patch embedding $\text{P}{\text{E}}_{i}^{sc}\in {\mathbb{R}}^{e}$ for the *i*-th patch ${\text{x}}_{i}^{sc}$ is thus:

(2) $$\text{P}{\text{E}}_{i}^{sc}=\text{CNN}\left({\text{x}}_{i}^{sc}\right)$$

#### 2) CONTEXTUALIZED PATCH EMBEDDINGS

The patch embeddings $\text{P}{\text{E}}^{sc}\in {\mathbb{R}}^{m\times e}$ are produced *independently* for each patch. In other words, these embeddings **PE**^*sc*^ do not encode inter-patch relationships. These embeddings **PE**^*sc*^ are fed to a transformer to learn inter-patch relationships. Similar to vision transformers [27], patch-wise sinusoidal positional embeddings $\text{PP}{\text{E}}^{sc}\in {\mathbb{R}}^{m\times e}$ are added to **PE**^*sc*^ to encode the position of input patches. The resultant embeddings are then fed to a transformer to produce contextualized patch embeddings $\text{CP}{\text{E}}^{sc}\in {\mathbb{R}}^{m\times e}$.


(3) $$\text{CP}{\text{E}}^{sc}=\text{Transformer}\left(\text{P}{\text{E}}^{sc}=\text{PP}{\text{E}}^{sc}\right)$$

These contextualized embeddings $\text{CP}{\text{E}}^{sc}\in {\mathbb{R}}^{m\times e}$ are then averaged along the *m*-dimension to produce an *e*-dimensional embedding vector ${\bar{\text{CPE}}}^{sc}\in {\mathbb{R}}^{e}.{\bar{\text{CPE}}}^{sc}$ encodes the local (from CNN) and global (from Transformer) information in an image **X**^*sc*^.

#### 3) CONTEXTUALIZED SCALE EMBEDDINGS.

The embedding ${\bar{\text{CPE}}}^{sc}$ encodes the information in an image **X**^*sc*^ at scale *sc*. Let us assume that we have S scales. For each $sc\in \left[0,\ldots ,S\right]S$, we produce embedding vector ${\bar{\text{CPE}}}^{sc}$ and concatenate them to produce scale-level embeddings $\text{SE}=\text{Concat}\left({\bar{\text{CPE}}}^{1},\ldots ,{\bar{\text{CPE}}}^{S}\right)$. These embeddings $\text{SE}\in {\mathbb{R}}^{S\times e}$ do not encode information about the relationships between the different scales. To learn scale-aware representations while retaining positional information about each scale, scale-level learnable positional embeddings $\text{PSE}\in {\mathbb{R}}^{sc\times e}$ are added^1^ to **SE**^*sc*×*e*^. The resultant embeddings are then fed to another transformer to produce contextualized scale embeddings $\text{CSE}\in {\mathbb{R}}^{S\times e}$.


(4) $$\text{CSE}=\text{Transformer}\left(\text{SE}+\text{PSE}\right)$$

For predicting the diagnostic class, ScATNet first flattens the scale-aware embeddings $\text{CSE}\in {\mathbb{R}}^{sc\times e}$ to produce a (*sc*·*e*)-dimensional vector and then classifies it using a linear classifier into *C* diagnostic categories.

### C. SOFT-LABELS FOR SKIN BIOPSY IMAGES

**FIGURE 3.**
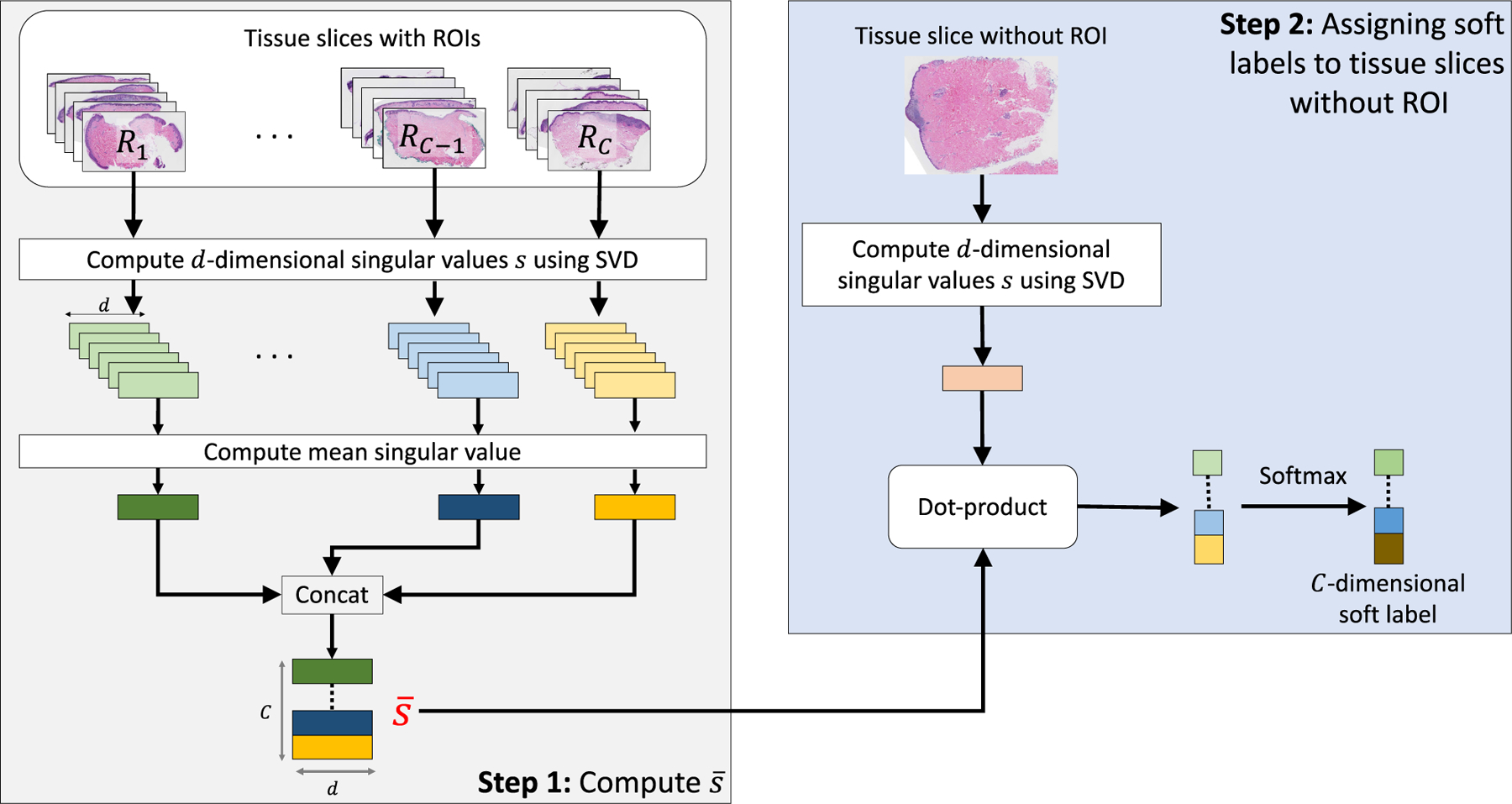
Overview of Soft labels calculation. Diagnostically constrained soft labels are calculated for tissue slices without an ROI using singular value decomposition (see Section III-C).

**FIGURE 4.**
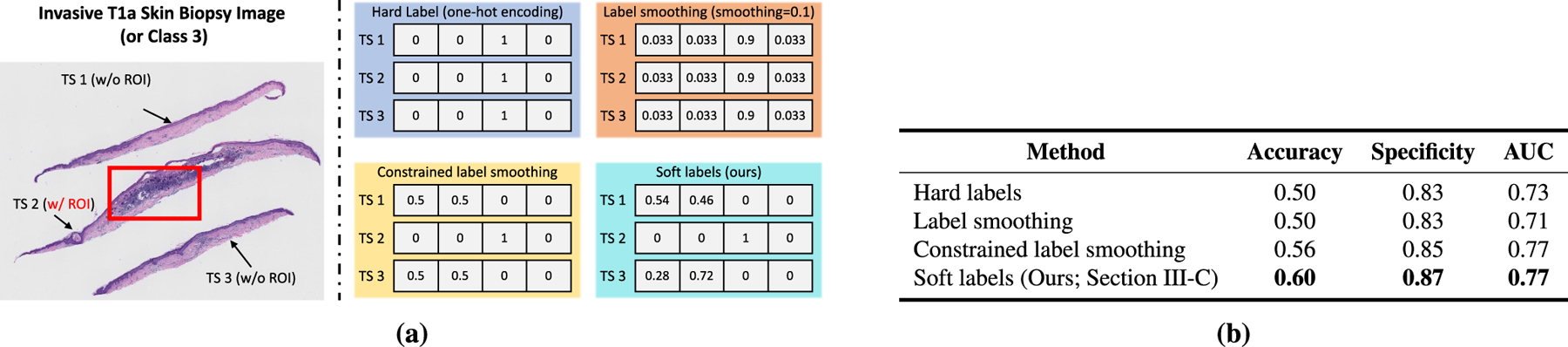
(a) shows different labeling methods, including our soft label method, for an *pT1a* skin biopsy image with three tissue slices and one representative region of interest (red box) that helped expert pathologists in diagnosing the image. (b) compares the performance of different labeling methods. Our soft labeling method is simple and effective; it reduces the ambiguity that arises during training because of multiple tissue slices in a WSI that do not have a ROI and helps improve the performance. In (b), we do not report sensitivity and specificity, because their values are the same as accuracy.

Skin biopsy images often contain multiple tissue slices on a single WSI, as shown in Figure 4. In general, the representative regions-of-interest (ROIs; shown in red in Figure 4) that helped pathologists in diagnosis belong to one or two tissue slices, while the other tissue slices may correspond to other diagnosis categories. Assigning the same diagnostic label to all tissue slices (similar to MIL-based approaches) results in more false tissue-label pairs and hinders learning representations. To address this, we propose a soft labeling method, as illustrated in Figure 3.

Given a dataset D with *N* training WSIs along with representative ROIs for each WSI (each WSI contains multiple slices) that helped in diagnosis, we aim to assign soft labels to tissue slices that do not have ROIs. Tissue slices from each WSI are extracted and then categorized into one of the two sets: (1) tissue slices R with an ROI and (2) tissue slices NR without an ROI. Since each slice in R has a representative ROI, we further split R into *C* subsets, $R=\left\{R_{1},\ldots ,R_{C}\right\}$, based on the diagnostic category, where *R*_*i*_ represents the subset for diagnostic category *i* and *C* denotes the number of diagnostic categories. Next, we compute the mean singular value vector ${\bar{\text{s}}}_{i}$ for each subset *R*_*i*_ as:


(5) $${\bar{\text{s}}}_{i}=\frac{1}{n}\sum_{j=1}^{n}{\text{s}}_{i}^{j}$$

where ${\text{s}}_{i}^{j}$ is the *d*-dimensional singular-value vector obtained after applying singular-value decomposition (SVD) to the *j*-th tissue slice in *R*_*i*_. The idea is to use these vectors to represent the appearance of the diagnostic categories. We used singular values because of their uniqueness and robustness properties [42]–[45]. However, other dimensionality reduction methods could also be used.

For the *j*-th slice in NR, the *C*-dimensional soft label vector ${\hat{y}}^{j}$ is computed as:

(6) $${\hat{y}}^{j}=\text{softmax}\left(\bar{\text{s}}\cdot {\hat{\text{s}}}^{j}\right)$$

where ${\hat{\text{s}}}^{j}$ is a *d*-dimensional singular value vector obtained after applying SVD to the *j*-th tissue slice in NR and $\bar{s}=\left\{{\bar{s}}_{1},\ldots ,{\bar{s}}_{C}\right\}$.

Tissue slices without an ROI do not help in the diagnosis decisions. Clinically, such slices can often belong to lower diagnostic categories than the category assigned to the WSI they are part of. We incorporate this diagnostic constraint in our soft labeling method. For a four-class dataset (1: *MMD*, 2: *MIS*, 3: *pT1a*, and 4: *pT1b*), suppose that a WSI corresponding to class *k* has *m* tissue slices and one of the tissue slices has an ROI, as shown in Figure 4. Soft label vectors ${\hat{y}}^{j}$ for the *j*th slices without ROI (*j* ∈ [0,*m* − 1]) can be obtained from equation 6. Then, to take one step further, *diagnostically constrained* soft label vector ${\tilde{y}}^{j}=\left\{{\tilde{y}}_{1}^{j},\ldots ,{\tilde{y}}_{C}^{j}\right\}$ is computed as:

(7) $${\tilde{y}}_{c}^{j}=\frac{{\hat{y}}_{c}^{j}}{\sum_{c=0}^{k}{\hat{y}}_{c}^{j}},\;if\;c<k\;{\tilde{y}}_{c}^{j}=0\;if\;c\geq k$$

Figure 4 illustrated an example WSI corresponding to class 3 (pT1a), which has three tissue slices, and one of the tissue slices has an ROI. If the soft label vectors ${\hat{y}}^{j}$ for these two slices without ROI are [0.46,0.39,0.08,0.07], [0.21,0.54,0.1,0.15], the resulting soft label vectors with the diagnostic constraint ${\tilde{y}}^{j}$ are [0.54,0.46,0,0], and [0.28,0.72,0,0] respectively.

## IV. EXPERIMENTAL RESULTS

### A. DATASET AND EVALUATION

#### 1) SKIN BIOPSY DATASET AND GROUND TRUTH CONSENSUS

The data used for this study was acquired as a part of the MPATH study (R01CA151306) and consists of 240 skin biopsy images with hematoxylin and eosin (H&E) staining [6]. The study was approved by the Institutional Review Board at the University of Washington with protocol number STUDY00008506. These biopsy images were interpreted by a consensus panel of three experienced dermatopathologists using the modified Delphi approach [46]. The consensus panel assessments were grouped into five different MPATHDx (Melanocytic Pathology Assessment Tool and Hierarchy for Diagnosis) [47] simplified categories based on perceived risk for progression. These five classes were regrouped to four diagnostic classes for the classification task in this paper due to limited sample size in Classes I and II and because the clinical risk for progression of both Class I and Class II is extremely low. The diagnostic terms we use for each class are as follows: 1) Class I-II: *mild and moderate dysplastic nevi* (**MMD**), which is very low risk to low risk, 2) Class III: *melanoma in situ* (**MIS**), which is higher risk than *MMD*, 3) Class IV: *invasive melanoma stage pT1a* (**pT1a**) which is higher risk for local/regional progression, and 4) Class V: *invasive melanoma stage* ≥*pT1b*(**pT1b**)which is the greatest risk for regional and/or distant metastases. We randomly split 240 WSIs into 102 training, 23 validation and 115 test WSIs (see Table 1). Additionally, the consensus panel of three experienced dermatopathologists marked in total 240 regions of interest (ROIs) that best defined the diagnostic classification of each case during the review process. Information about these ROIs was used to produce soft labels for the training set (Section III-C).

#### 2) OUTCOME METRICS

The performance of ScATNet is evaluated in terms of the following standard quantitative metrics: (1) classification (or Top-1) accuracy, (2) F1 score, (3) sensitivity, (4) specificity, and (5) area under receiver operating characteristic curves (ROC-AUC). The values of these metrics range between zero and one, and higher values of these metrics mean better performance. Multi-class F1 and specificity have the same value as accuracy.

#### 3) ACCURACY DATA FROM U.S. PATHOLOGISTS

To compare the results from ScATNet with the interpretations of practicing U.S. pathologists, we used data from a prior clinical study in which 187 pathologists interpreted the same WSIs [6]. Each pathologist interpreted a random subset of 36 cases, and their diagnoses were classified into the same four diagnostic categories. This resulted in 10 independent diagnostic labels (on an average) per slide and provided a way to compare the classifications performed by human pathologist to ScATNet. These interpretations are only used for independent evaluation. The ground truth diagnosis of each slide is the consensus diagnosis of three experienced dermatopathologists.

### B. IMPLEMENTATION DETAILS

#### 1) EXTRACTING TISSUE SLICES FROM WSIs

The original WSIs were collected at a zoom level of 40×. Because WSIs at 40× require extensive computational resources, we extracted WSIs at lower zoom levels of 7.5× (average size 8348 × 7202), 10× (average size 11130 × 9603), and 12.5× (average size 13913 ×12003). These zoom levels were selected based on previous work on histopathological image classification for different tissues [11], [16], [40], since they provide a good tradeoff for 1) capturing sufficient local context without including irrelevant details and 2) providing variable local information without losing similar correlation. We refer to different zoom levels as ‘‘input scales’’ in this work. Each WSI has multiple tissue slices with a background region between the slices that does not aid in diagnosis (Figure 4). Therefore, individual tissue slices were extracted using a histogram-based segmentation method of Otsu [48] followed by morphological operations (opening-closing and hole filling) and contour-related operations available in OpenCV.

#### 2) SOFT-LABELS

To assign soft labels for tissue slices without an ROI, SVD is applied to obtain *d*-dimensional singular-value vectors as described in the Methods section. In this study, *d* is set to 50.

##### a: ARCHITECTURE

We use MobileNetv2 [49] pretrained on the ImageNet dataset [41] as our CNN for extracting patch-wise embeddings. MobileNetv2 was chosen, because it is light-weight, fast, and delivers state-of-the-art performance across different machine vision tasks, such as classification, detection, and segmentation. ScATNet is not limited to a particular CNN and other CNNs, such as VGG [50] and ResNet [10] may also be suitable for extracting patch-wise embeddings.

MobileNetv2outputs1280-dimensionalpatch-wiseembeddings after global average pooling. ScATNet projects these patch-wise embeddings linearly to a 128-dimensional space (*e* = 128) and then learns contextualized patch-wise and scale-wise embeddings using transformers. For learning contextualized patch-wise and scale-wise representations, a stack of two transformer units is used. Also, in each transformer unit, the number of heads in the self-attention layer is set to 4, and the feed forward network dimension is set to 512.

### C. TRAINING DETAILS

ScATNet is trained for 200 epochs in an end-to-end fashion using the ADAM optimizer with a linear learning rate warm-up strategy and step learning rate decay. The learning rate is first warmed up from 10^−6^ to 5 × 10^−4^ in 500 steps. In the next 50 epochs, the model is trained with a learning rate of 5 × 10^−4^. After that, the learning rate is reduced by half at the 100-th and 150-th epochs. Because of the large size of these images, extensive computational resources are required. To learn representations with limited computational resources, we freeze the convolutional layers in a CNN and train only the transformer networks. Our models are trained on a single NVIDIA GeForce 2080 GPU with 10 GB GPU memory. Similar to other medical imaging datasets, our dataset is small. Therefore, to improve its robustness against stochastic noise, we average best 3 and best 5 model checkpoints within a single training process [51] and select the one that performs best on the validation set. We then evaluate it on the (unseen) test set. A WSI in a test set may contain multiple tissue slices. To predict the final diagnostic label, we use max-voting. This choice is inspired by pathologists’ diagnosing behavior, i.e., if one of the tissue slices in a WSI is invasive melanoma, then the entire WSI corresponds to invasive melanoma and cannot be *MMD* or *MIS*.

### D. BASELINE METHODS

ScATNet’s performance is compared with five recent whole slide image classification methods.

#### 1) PATCH-BASED CLASSIFICATION

The first method is a standard patch-based CNN classification framework that was built following saliency-based methods, related to the work of Hou *et al.* [11] and that of Mercan *et al.* [39], (R1 and R2 in Table 2). This method treats each patch independently and assigns the same diagnostic label to all patches in the WSI during training. During evaluation, majority-voting is used for predicting the slide-level diagnostic label. Similar to the use of ScATNet, Mobilenetv2, pretrained on the ImageNet dataset was used as the CNN model.

#### 2) WEIGHTED FEATURE AGGREGATION

The second method is a CNN-based deep feature extraction framework developed by Mercan *et al.* [15] that builds slide-level feature representations via weighted aggregation of the patch representations (R3 and R4 in Table 2). Under this framework, feature extraction is performed in three steps: (1) using a CNN (e.g. VGG16) to extract features on a patch-by-patch basis; (2) concatenating the weighted instances of the extracted feature activations using either penultimate layer features (penultimate-weighted) or hypercolumn features (hypercolumn-weighted) to form patch-level feature representations; and (3) fusing the patch-level representations via average pooling to form the slide-level representation.

#### 3) ChikonMIL

The method of Chikontwe *et al.* (ChikonMIL) (R3 in Table 2) [7] first selects the top-k patches, and then uses these patches for instance- and bag-representation learning. This method also uses a center loss that reduces intra-class variability and a soft assignment to learned diagnostic centroid for final diagnosis.

#### 4) MS-DA-MIL

Multi-scale Domain-adversarial Multiple-instance (MS-DA-MIL) CNN developed by Hashimoto *et al.* [8] (R7 and R8 in Table 2) is a framework that learns from groups of patches extracted at different scales (x10 and x20) with attention mechanism. However, in contrast to the proposed end-to-end learning framework, MS-DA-MIL-CNN first trains a single-scale MIL network to classify for each scale. Then, a multi-scale network is trained using the features extracted using pre-trained single-scale MIL networks.

#### 5) STREAMING CNN

Streaming CNN is a work of Pinckaers *et al.* [22] (R4 in Table 2). This method uses a patch-based approach with gradient checkpointing and streaming, which allows it to classify whole slide images in an end-to-end fashion.

### E. RESULTS

#### 1) HARD vs. SOFT LABELS

The performance of our soft labeling method (Section III-C) is compared with three other labeling methods. For illustration, for the four classes in our dataset (1: *MMD*, 2: *MIS*, 3: *pT1a*, and 4: *pT1b*), we use a WSI corresponding to *pT1a* (class 3; shown in Figure 4) with 3 slices, one having a ROI.

- *Hard labels:* Similar to MIL-based approaches, all tissue slices in the WSI are assigned the same diagnostic label. For the above example, each tissue slice will have a label of [0, 0, 1, 0] (one-hot vector encoding).
- *Label smoothing:* The label smoothing method of Szegedyet *et al.* [52] produces soft labels that are a weighted average of the hard labels and the uniform distribution over labels. It regularizes the network and helps improve the performance [53]. For the same example, the soft labels for each of these slices would be [0.033, 0.033, 0.9, 0.033] with a label smoothing value of 0.1. In other words, the label for class 3 is smoothed from 1 to 0.9 and the remaining mass of 0.1 is equally distributed among the remaining three classes.
- *Constrained label smoothing:* This extends the hard labels and label smoothing methods by incorporating the diagnostic constraint that tissue slices without a ROI should belong to lower diagnostic categories. For example, if the WSI has a hard label of *pT1a* (i.e. class 3), then the tissue slices without a ROI can only belong to lower diagnostic categories (i.e., *MMD* and *MIS*). For the same example as above, the slice with an ROI will have a label of [0, 0, 1, 0] while the slices without an ROI will have constrained labels of [0.5, 0.5, 0, 0].

Figure 4a contrasts our soft labeling method with these methods while quantitative comparison between these methods is given in Figure 4b. These experiments demonstrated that our soft labeling method is more effective as compared to these existing methods. In subsequent experiments, we use our soft labeling method.

##### a: IMPACT OF NUMBER OF PATCHES m

**FIGURE 5.**
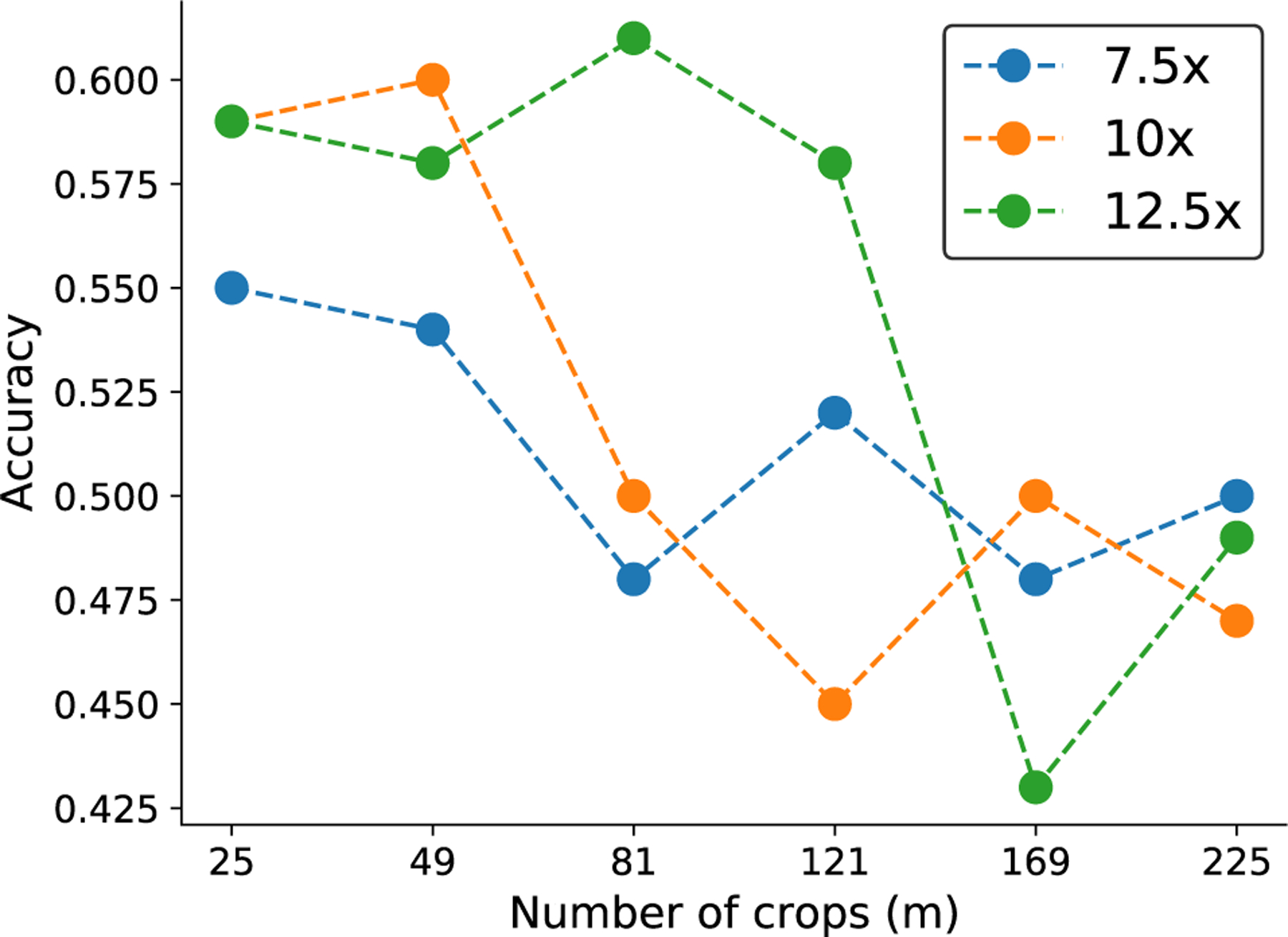
Effect of number of crops (*m*) on the performance of ScATNet (single scale) for inputs at three different scale levels (7.5x, 10x, and 12.5x).

Figure 5 compares the performance of single scale ScATNet with different numbers of crops *m* at three different input resolutions (7:5×, 10×, and 12:5×). Using fewer crops at larger resolution (e.g., 25 crops at a resolution of 12:5×) and more crops at smaller resolutions (e.g., 81 crops at a resolution of 7:5×) hurts the performance. This is likely because MobileNetv2, the CNN used in this work, is pre-trained on the ImageNet dataset at a fixed image size of 224 × 224. With very large (fewer number of crops at larger image resolution) or very small (larger number of crops at smaller image resolution) patch sizes, the CNNs may have difficulty in capturing representative features and yield poor patch embeddings, which hurts the performance. We note that scaling patch size alone may not be an optimal solution and future studies, especially compound model scaling in EfficientNet [54], may help improve the performance.

In the rest of the experiments, we used *m* = 25 for 7.5× input resolution, *m* = 49 for 10× input resolution, and *m* = 81 for 12.5× input resolution, as these had the best performance.

##### b: Single vs. MULTIPLE INPUT SCALES

**FIGURE 6.**
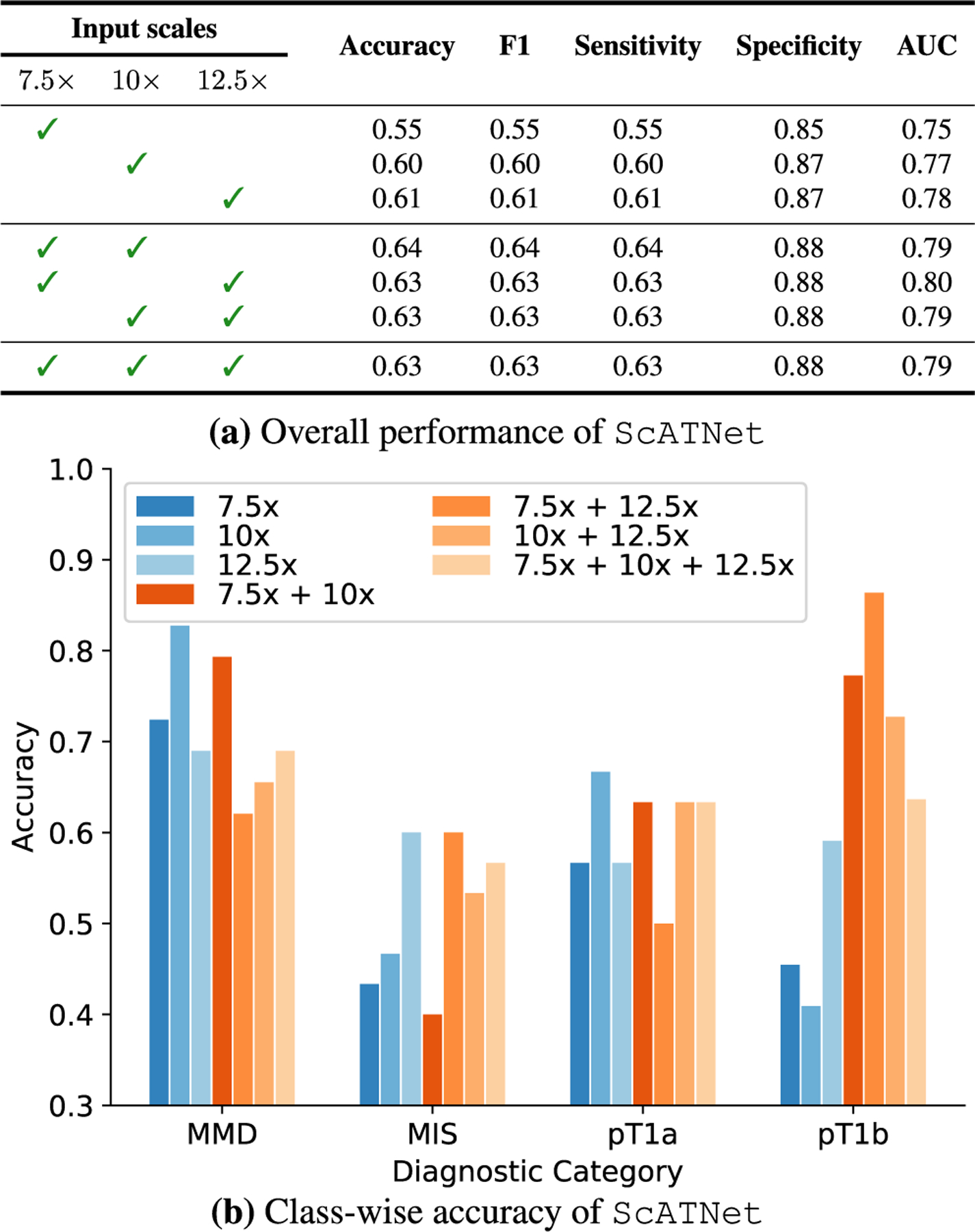
Effect of single and multiple input scales. For single and multiple input scales, we compared the overall performance of ScATNet across different metrics in (a) while in (b), we compared the class-wise accuracy. With multiple input scales, overall and class-wise performance, especially in invasive cancer categories (pT1a and pT1b), of ScATNet improved across all evaluation metrics. Diagnostic terms are defined as the following: *mild and moderate dysplastic nevi* (*MMD*), *melanoma in situ* (*MIS*), *invasive melanoma stage pT1a* (*pT1a*), *invasive melanoma stage ≥pT1b* (*pT1b*).

Figure 6a compares the overall performance of ScATNet across different metrics on single- and multi-scale inputs, while class-wise accuracy is given in Figure 6b. With inputs at multiple scales, we observe improvements in overall as well as class-wise performance. Notably, we observe significant improvement with multiple scales (two and three scales) in the *pT1b* invasive melanoma cancer category. Compared to two scales, the overall performance with three scales remains the same. However, with three scales, the performance across all diagnostic classes (Figure 6b) is much more evenly distributed, which is not seen in all other combinations.

##### c: COMPARISON WITH BASELINE METHODS

**FIGURE 7.**
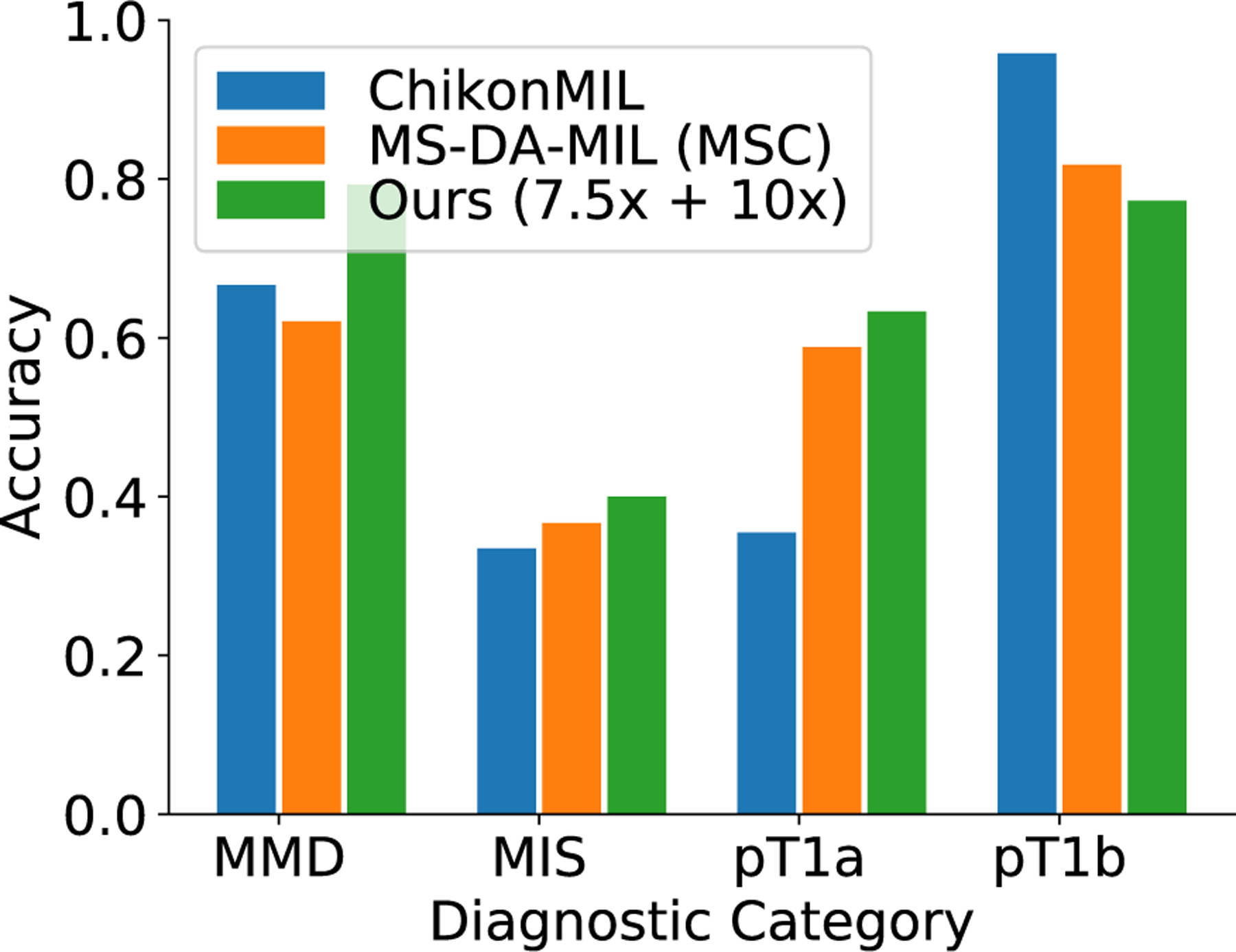
Comparison of class-wise accuracy with state-of-the-art WSI classification methods on the test set. Diagnostic terms are defined as the following: *mild and moderate dysplastic nevi* (*MMD*), *melanoma in situ* (*MIS*), *invasive melanoma stage pT1a* (*pT1a*), *invasive melanoma stage ≥pT1b* (*pT1b*). Overall, ScATNet delivered better performance across all diagnostic categories except the pT1b category.

Figure 2 compares the classification performance of ScATNet with existing methods on the test set. ScATNet outperforms all five existing methods to which it was compared by a significant margin across different metrics. Furthermore, compared to the ChikonMIL method [7] and the MS-DA-MIL method [8] with multi-scale input, which delivered the two best performances among the five baseline methods, ScATNet delivered better performance across all diagnostic categories (see Figure 7), except the pT1b category. This is likely because the ChikonMIL method samples more relevant patches corresponding to the pT1b category as compared to other diagnostic categories, while the MS-DA-MIL method uses an input at higher resolution (x20), which might yield more information at the cellular level that helped to distinguish the pT1b category. We believe that complementing the proposed method with the patch sampling method of Chikontwe *et al.* (2020) would further improve the performance. We will investigate such methods in the future.

##### d: COMPARISON WITH U.S. PATHOLOGISTS

Table 3 shows that ScATNet achieves similar performance to practicing U.S. pathologists who interpreted these same cases in overall accuracy (pathologists vs. ScATNet: 0.65 vs. 0.64), suggesting its potential as a second reader to help pathologists in clinical settings for reducing classification uncertainties.

## V. DISCUSSION

Previous studies on computer-aided skin lesion analysis have been mainly focused on using dermoscopic images due to its inexpensiveness and availability [55]–[57]. Although dermoscopic images showed improvement for diagnosis of skin cancer compared to bare visual inspection, the gold standard for the diagnosis of melanocytic lesions is the interpretation of histopathology slides. There has been limited application of deep learning techniques in whole slide skin biopsy images due to their gigapixel size and the lack of large public datasets. Earlier studies analyzing whole slide skin biopsy images using deep learning have focused on dermis and epidermis segmentation, as well as two- or three-class classification problems. For example, Phillips *et al.* [58] explored segmentation of dermis and epidermis as well as tumor segmentation using convolutional neural network with a dataset of 50 WSIs (Training/validation/test: 36/7/7). Hekler *et al.* [59], [60] studied the binary classification of *nevi* vs. *melanoma* with a dataset of 695 WSIs (Training/Test: 595/100). Similarly, Lu and Mandal [61] and Xu *et al.* [17] 17 melanocytic nevi, and 32 superficial spreading melanoma) performed a three-way classification task (17 normal skin, using 66 WSIs. Note that the dataset used by Lu and Mandal *et al.* [61] and Xu *et al.* [17] is much smaller than ours and limited to only two of our classes, making direct comparison impossible.

Unlike these studies, this work classifies the full spectrum of melanocytic skin biopsy lesions ranging from mildly atypical nevi and more advanced atypical pre-cursor lesions, to melanoma in situ to invasive melanoma. Our dataset consists of 240 WSIs, including 115 WSIs in an independent test set (Table1). An independent test set allows us to demonstrate the generalization ability of ScATNet. A key strength of our work is that we were able to compare the diagnostic classification of ScATNet with the performance of actively practicing U.S. pathologists who interpreted the same cases (test set) in an independent study.

Although the proposed method has shown great potential for automated melanocytic lesion classification, limitations are recognized. Our study is only relevant to melanocytic lesions, while only about one in four skin biopsies have melanocytic cells [62]. Moreover, despite having an independent test set, ScATNet was evaluated on only 115 WSIs. In order to demonstrate its application in clinical settings, ScATNet should be tested on a larger test set. Also, in this paper, we only studied skin biopsies. However, we believe that ScATNet is generic and can be extended to other types of biopsy images, such as breast and lung.

## VI. CONCLUSION

Diagnosis of melanocytic lesions is among the most challenging areas of pathology. Previous studies indicate that diagnostic errors occur frequently [3]–[5]. False positive readings for suspected melanoma range from 6% to 17% [63], [64]. Diagnostic errors may lead to inappropriate treatment decisions and harm to patients. With FDA approval, digitized whole slide imaging systems show great potential for improving the diagnostic performance of pathologists. In this paper, we introduce the scale-aware transformer network ScATNet for learning representations from variably-sized whole slide skin biopsy images at multiple scales. Compared to existing methods, ScATNet delivered better performance. Importantly, ScATNet also delivered comparable performance to practicing U.S. pathologists who interpreted the same cases. The implementations of the models we use and algorithms we introduce are available at https://github.com/meredith-wenjunwu/ScATNet.

## Figures and Tables

**FIGURE 8.**
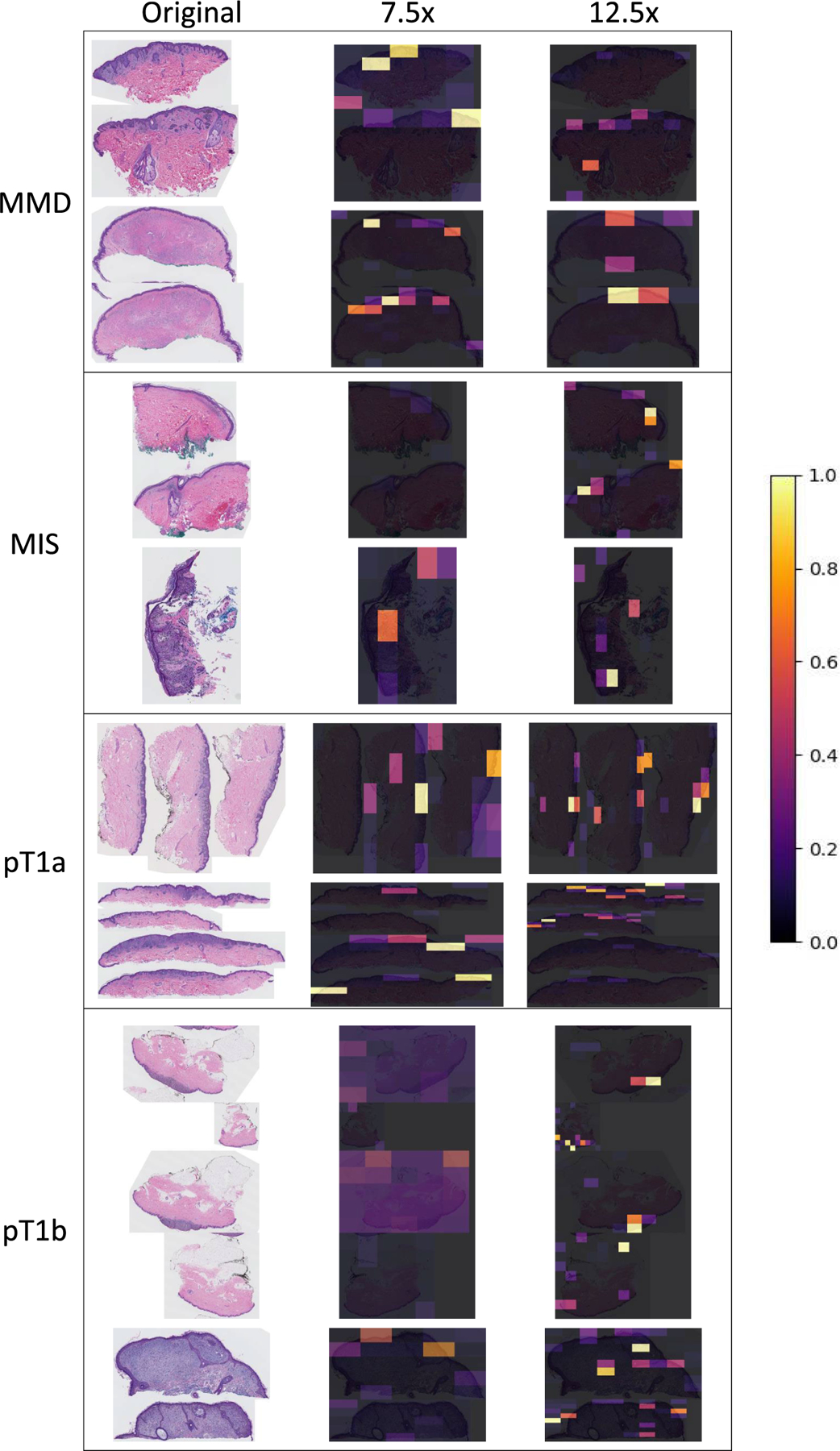
Visualization of gradient in ScATNet. The left column shows original whole slide images in all diagnostic categories: *mild and moderate dysplastic nevi* (*MMD*), *melanoma in situ* (*MIS*), *invasive melanoma stage pT1a* (*pT1a*), *invasive melanoma stage ≥pT1b* (*pT1b*). The right two columns are the corresponding gradient maps calculated from 7.5x and 12.5x input scales. All examples shown were correctly classified into their diagnostic categories. Colors from purple to yellow are assigned to values between 0 and 1.

**FIGURE 9.**
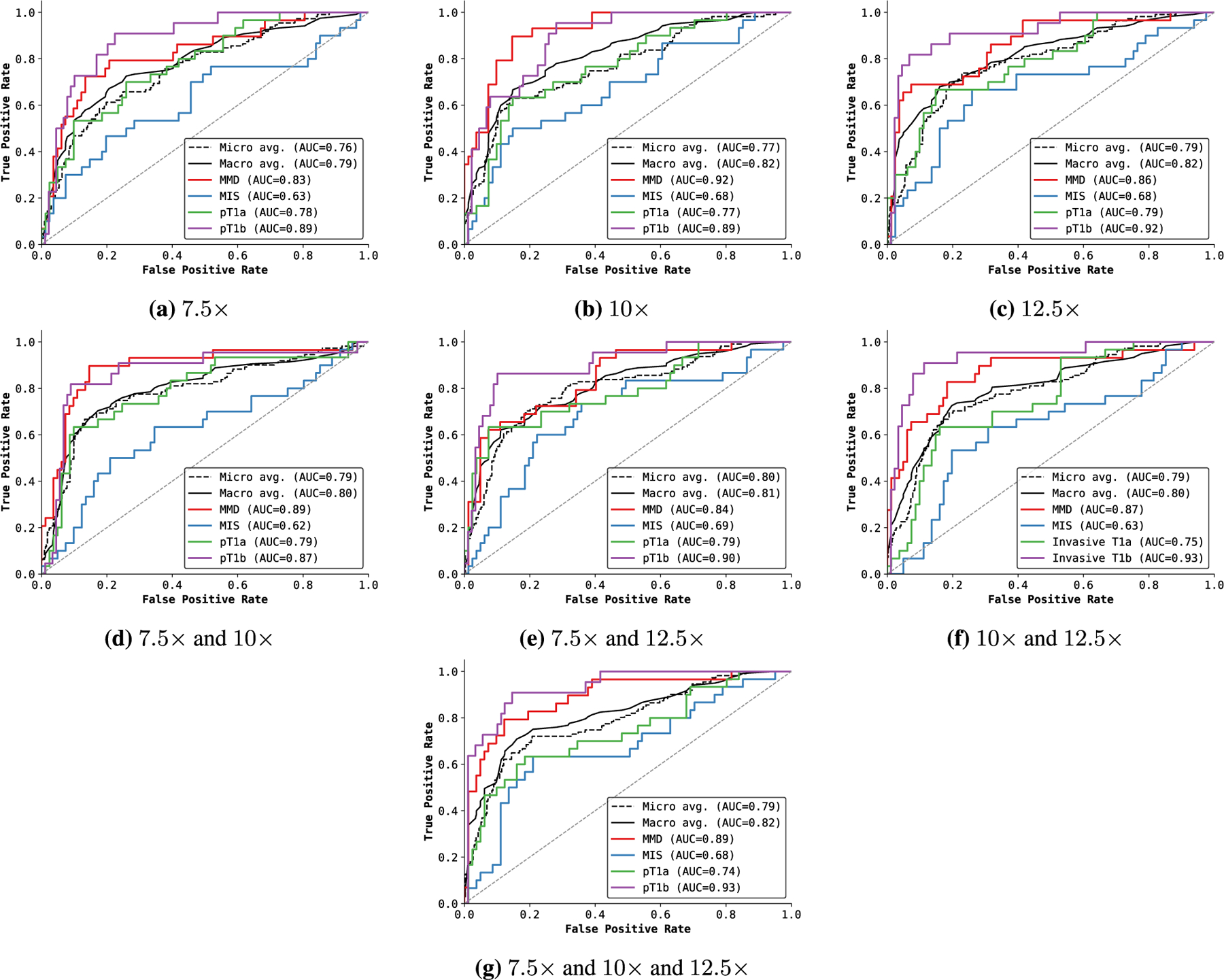
Receiver operating characteristic (ROC) curves of ScATNet with different numbers of input scales. For a single scale (a-c), the performance improves with the input scale, especially for invasive cancers. For two scale combinations (d-f), we do not observe significant gains. However, a combination of smaller and larger input scales (7.5x and 12.5x) delivered good performance across all diagnostic classes. Diagnostic terms are defined as the following: *mild and moderate dysplastic nevi* (*MMD*), *melanoma in situ* (*MIS*), *invasive melanoma stage pT1a* (*pT1a*), *invasive melanoma stage* ≥*pT1b* (*pT1b*).

**FIGURE 10.**
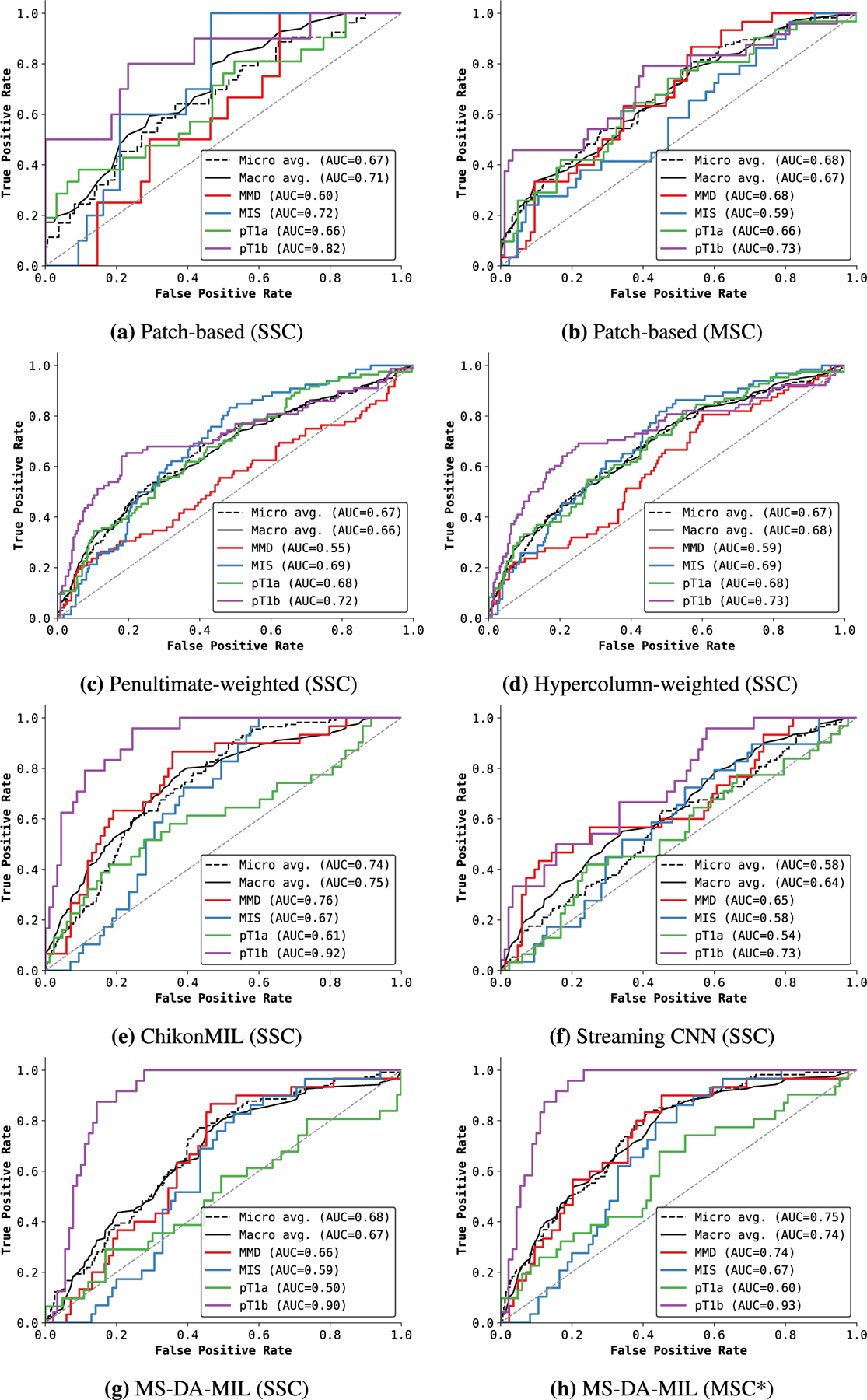
Comparison of ROC curves with state-of-the-art WSI classification methods on the test set. Here, SSC denotes single input scale (10 ). MSC denotes multiple input scales (7.5x, 10x, 12.5x), while MSC* denotes 10x, 20x. Overall, the MS-DA-MIL method of Hashimoto *et al.* [8] delivers the best performance of all other existing methods. Diagnostic terms are defined as the following: *mild and moderate dysplastic nevi* (*MMD*), *melanoma in situ* (*MIS*), *invasive melanoma stage pT1a* (*pT1a*), *invasive melanoma stage ≥pT1b* (*pT1b*).

**TABLE 1.** Statistics of skin biopsy whole slide image (WSI) dataset. The average WSI size is computed at a magnification factor of 10x. Diagnostic terms for the dataset used in this study are as follows: *mild and moderate dysplastic nevi* (MMD), *melanoma in situ* (MIS), *invasive melanoma stage pT1a* (pT1a), *invasive melanoma stage ≥pT1b* (pT1b).

Diagnostic Category	Number of WSIs				Average WSI size (in pixels)
	Training	Validation	Test	Total	
MMD	26	6	29	61	11843 × 10315
MIS	25	5	30	60	9133 × 8501
pT1a	33	6	34	73	9490 × 7984
pT1b	18	6	22	46	14858 × 12154

Total	102	23	115	240	11130 × 9603

**TABLE 2.** Comparison of overall performance with state-of-the-art WSI classification methods across different metrics on the test set. Here, SSC denotes single input scale (10). MSC denotes multiple input scales (7.5, 10, 12.5). MSC* denotes multiple input scales (10, 20).

Row #	Method	Accuracy	Fl	Sensitivity	Specificity	AUC
R1	Patch-based (SSC)	0.35	0.35	0.35	0.79	0.67
R2	Patch-based (MSC)	0.40	0.40	0.40	0.80	0.68
R3	Penultimate-weighted (SSC)	0.44	0.44	0.44	0.81	0.67
R4	Hypercolumn-weighted (SSC)	0.43	0.43	0.43	0.81	0.67
R5	Streaming CNN (SSC)	0.32	0.32	0.32	0.77	0.58
R6	ChikonMIL (SSC)	0.56	0.56	0.56	0.85	0.74
R7	MS-DA-MIL (SSC)	0.49	0.49	0.49	0.83	0.68
R8	MS-DA-MIL (MSC*)	0.58	0.58	0.58	0.86	0.75

R9	ScATNet (SSC)	0.60	0.60	0.60	0.87	0.77
R10	ScATNet (MSC)	**0.64**	**6.64**	**0.64**	**0.88**	**0.79**

**TABLE 3.** Comparison of ScATNet with pathologists’ (PG) performance. Pathologists’ performance data is from a prior *independent* clinical study of 187 pathologists [6] who interpreted these same 115 cases in our test set (Table 1). Diagnostic terms are defined as the following: *mild and moderate dysplastic nevi* (*MMD*), *melanoma in situ* (*MIS*), *invasive melanoma stage pT1a* (*pT1a*), *invasive melanoma stage ≥pT1b* (*pT1b*).

Diagnostic Category	Accuracy		F1		Sensitivity		Specificity	
	PG	Ours	PG	Oars	PG	Ours	PG	Ours
MMD	0.92	0.79	0.71	0.75	0.92	0.79	0.76	0.89
MIS	0.46	0.40	0.49	0.44	0.46	0.40	0.85	0.84
pT1a	0.51	0.65	0.62	0.63	0.51	0.65	0.95	0.84
pT1b	0.72	0.77	0.72	0.74	0.78	0.77	0.97	0.92

Overall	0.65	0.64	0.65	0.64	0.65	0.64	0.88	0.88

## Footnotes

1 Unlike the number of patches *m*, the number of scales S is fixed. Therefore, we learned the positional embeddings for each scale using torch.nn.Embedding in PyTorch. Compared to sinusoidal positional embeddings, learned embeddings improve the performance by about 0.5–1.0%.

## APPENDIX

### A. OUTCOME METRICS

The following metrics were used to evaluate the performance of ScATNet [65]:

- Classification (or Top-1) accuracy counts the number of times the predicted label is the same as the ground truth label and is defined as:
  $$\text{Accuracy}=\frac{\text{TP}}{\text{TP}+\text{FP}+\text{TN}+\text{FN}}$$
  where TP, FP, TN, and FN denotes the true positive, false positive, true negative, and false negatives respectively.
- F1-score is a harmonic mean of precision *P* and recall *R* and is defined as:
  $$\text{F}1\text{-score}=\frac{2PR}{P+R}$$
  where $P=\frac{\text{TP}}{\text{TP}+\text{FP}}$ and $R=\frac{\text{TP}}{\text{TP}+\text{FN}}$.
- Sensitivity measures proportion of the positive cases that are correctly classified and is defined as:
  $$\text{Sensitivity}=\frac{\text{TP}}{\text{TP}+\text{FN}}$$
- Specificity measures the proportion of the negative cases that are correctly classified and is defined as:
  $$\text{Specificity}=\frac{\text{TN}}{\text{TN}+\text{FP}}$$
- Area under receiver operating characteristics curve (ROC-AUC) is a graph obtained by varying the threshold for diagnostic decision, illustrating the discrimination ability of the classifier. We use a One-vs-rest scheme, which computes the AUC of each class against the rest [66].

The values of these metrics range between zero and one, and higher values of these metrics mean better performance.

### B. SALIENCY ANALYSIS

Saliency analysis using gradients helps identify relevant areas in an input image that contributed to the prediction [67]. Figure 8 shows that both 7.5× and 10× contributed to the decision in the cases of *MMD* and *pT1a*, while 12.5× contributes more in the cases of *MIS* and *pT1b*. This pattern illustrates that depending on the input whole slide image, diagnosis-specific features exist at different input scales and ScATNet learns to weigh these features automatically.

### C. ROC CURVES

In Figure 9, we compared the Receiver Operating Characteristic (ROC) curves of the proposed method with different numbers of input scales. With a single scale, the overall area under the curve (AUC) score as well as the class-wise AUC score of invasive cancer categories (*pT1a* and *pT1b*) improve with larger input scale. With two scales, we observed the best performance in the combination of the smallest and the largest scale (7.5× and 12.5×).

#### e: COMPARISON OF BASELINE METHODS

In Figure 10, we compared ROC curves of the baseline methods. The MS-DA-MIL method of Hashimoto *et al.* [8] delivered the best AUC score, compared to the weighted feature aggregation method by Mercan *et al.* [15], ChikonMIL method by Chikontwe *et al.* [7], the patch-based classification method [11], [39] and the Streaming CNN method [22]. With multiple input scales, the patch-based method did not show significant improvement in AUC score, but the performance across all classes is more evenly distributed.
